# Effects of Surface Pretreatments on Bond Strength and Morphology of Aprismatic Enamel

**DOI:** 10.3290/j.jad.b3240701

**Published:** 2022-10-18

**Authors:** Allegra Comba, Andrea Baldi, Martina Garavelli, Tatjana Maravic, Lorenzo Breschi, Annalisa Mazzoni, Claudia Mazzitelli, Nicola Scotti

**Affiliations:** a Researcher, Department of Surgical Sciences, Dental School Lingotto, University of Turin, Turin, Italy. Wrote original draft, supervision.; b PhD Student, Department of Surgical Sciences, Dental School Lingotto, University of Turin, Turin, Italy. Sample preparation and SEM analysis, supervision.; c Private Practitioner, Turin, Italy. Sample preparation and SEM analysis.; d Researcher, Department of Biomedical and Neuromotor Sciences, DIBINEM, University of Bologna – Alma Mater Studiorum, Bologna, Italy. Data curation, methodology, reviewed and edited manuscript.; e Full Professor, Department of Biomedical and Neuromotor Sciences, DIBINEM, University of Bologna – Alma Mater Studiorum, Bologna, Italy. Supervision, reviewed and edited manuscript.; f Associate Professor, Department of Biomedical and Neuromotor Sciences, DIBINEM, University of Bologna – Alma Mater Studiorum, Bologna, Italy. Supervision, reviewed and edited manuscript.; g Researcher, Department of Biomedical and Neuromotor Sciences, DIBINEM, University of Bologna – Alma Mater Studiorum, Bologna, Italy. Formal analysis and statistics, reviewed and edited manuscript.; h Associate Professor, Department of Surgical Sciences, Dental School Lingotto, University of Turin, Turin, Italy. Supervision, Project administration, Conceptualization.

**Keywords:** aprismatic enamel, adhesion, no-prep restorations, airborne-particle abrasion, polishing powders.

## Abstract

**Purpose:**

To evaluate the effect of different pretreatment protocols and adhesives on the shear bond strength and surface morphology of aprismatic enamel.

**Materials and Methods:**

Human maxillary incisors (N = 120) were assigned to five different groups according to pretreatment: 1) no treatment; 2) glycine; 3) sodium bicarbonate; 4) Al_2_O_3_ and 5) extra-fine bur. Then the teeth were divided into three subgroups, according to the adhesive applied: 3-step etch-and-rinse adhesive (ER), universal adhesive in ER mode, and universal adhesive in self-etch (SE) mode. Shear bond strength (SBS) testing was performed with a universal testing machine. For SEM observation, fifteen human molars were collected and analyzed after pretreatment with/without etching with 37% H_3_PO_4_ for 30s.

**Results:**

Al_2_O_3_ showed higher SBS than all other groups considered. Comparable SBS was obtained for other pretreatments. Universal adhesive in E&R mode performed better than did 3-step E&R and universal adhesive in SE mode. SEM images showed visible differences in enamel surface roughness.

**Conclusions:**

Airborne-particle abrasion with Al_2_O_3_ followed by etching with H_3_PO_4_ increased SBS on aprismatic enamel. The combination of airborne-particle abrasion with alumina powder followed by 15 s of H_3_PO_4_ etching and application of a universal adhesive in E&R mode proved to be the most effective adhesive protocol.

Over the past few decades, the development of new materials and different operative protocols has increased interest in adhesive and minimally invasive dentistry, which have influenced modern dental practices considerably.^11,34^ Indeed, the new approach is more conservative and fundamentally relies on the effectiveness of the chemomechanical connection between resin materials, enamel-dentin adhesives, and dental tissue.^37^ It is employed not only to treat early-stage dental pathologies, but also to obtain and modify smile esthetics (“no-prep” or “prepless” approach) without compromising the mechanical structure of the tooth.^22,39^

The formation of a durable interface between adhesives and dental substrates is achieved using the etch-and-rinse (ER) or the self-etch (SE) bonding approach.^10,35^ The ER approach requires conditioning the dental substrate and removing the smear layer with an acid (usually 37% orthophosphoric acid [H_3_PO_4_]), followed by rinsing with water. Meanwhile, the SE technique, also known as the etch-and-dry technique, is employed for the maintenance and resin infiltration of the smear layer, which slightly demineralizes thanks to the acidity of the adhesive itself.^7^ Recently, a new category of adhesives – universal adhesives – was introduced. Single-bottle universal adhesives can be used with either the ER or SE approach; moreover, they contain additional functional monomers that allow their application on several other substrates and are considered to be less subject to operator experience.^7^

Both the ER and SE approaches have been widely investigated. Studies agree that while adhesion to enamel is predictable and strong (especially when applied using the ER approach), due to the characteristics of substrates, adhesion to dentin is more sensitive to operator experience and dentin moisture.^24,28^ Furthermore, the ability of resin to infiltrate enamel and dentin is correlated with surface wettability and the substrate’s surface free energy, which are directly proportional to the degree of mineralization. This correlation explains the higher predictability of adhesion to enamel than to dentin.^36^

From a histological point of view, two different types of enamel are treated with adhesive materials: aprismatic and prismatic enamel. Aprismatic enamel is the superficial, irregular layer of enamel, averaging approximately 30 μm in thickness, without individual enamel rods or prisms due to the parallel arrangements of the crystallites. It is mostly found in deciduous teeth, but it has also been observed in different regions of permanent teeth.^21^ This tissue is made of inorganic substance and contains a form of hydroxyapatite that is no longer organized in prisms.^42^ Conversely, prismatic tissue is a deeper layer of enamel, comprised of 97% inorganic substance and hydroxyapatite and 3% water and organic components. Hydroxyapatite makes up almost the entire internal structure of this tissue, and it generates the enamel prisms which are surrounded by interprismatic enamel; this is what the literature refers to when discussing bond strength.^4^

Although bonding to prismatic enamel is considered predictable, easily reproducible and durable, several authors have expressed their concerns regarding the possibility of bonding to non-prepared and superficial enamel, a non-homogeneous layer consisting of areas that are not easily or evenly etchable.^1,33,38^ To overcome this problem, especially in the orthodontic field where bonding to intact enamel is an everyday occurrence, airborne-particle abrasion of the surface prior to bracket placement has been suggested.^26^ Moreover, in restorative dentistry, certain clinical protocols suggest applying prophylactic polishing powder (eg, glycine, bicarbonate, erythritol) and an extra-fine bur before the adhesive procedure to remove superficial biofilm and prepare the aprismatic enamel for additive reconstructions. However, no comparative studies exist about the influence of various aprismatic enamel pretreatment procedures in association with different adhesives on bond strength and surface morphology. Hence, precise clinical recommendations are lacking.

Accordingly, the aims of the present in-vitro research were 1. to evaluate the effects of different micro-abrasive powders and adhesives on the shear bond strength (SBS) of intact aprismatic enamel and 2. to evaluate the effects of different pretreatments on the superficial enamel surface. The null hypotheses tested were that 1. the type of surface pretreatment produced no differences in bond strength and morphological characteristics of the enamel; 2 the adhesive employed did not influence the final adhesive outcomes.

## MATERIALS AND METHODS

### Shear Bond Strength Test (SBS)

Sample size calculation was determined using G*Power 3.1.9.7 for Windows.^12^ Accordingly, 120 intact human permanent maxillary incisors, extracted for periodontal reasons and without carious lesions, demineralization, abrasions, cracks, or signs of wear of the enamel, were collected. The study was approved by the local ethics committee of the Dental School, University of Turin (DS_2019_016). The roots were removed at the dentin-enamel junction using a low-speed diamond saw under water cooling (Micromet, Remet; Bologna, Italy). Then, the tooth crowns were embedded in acrylic resin, with the flat buccal surface exposed, and stored in distilled water at 37°C until use.

The embedded specimens were randomly assigned to the following groups according to aprismatic enamel surface pretreatment (N = 24):

- Group 1 (G1): surface cleaning with a soft rotary brush under water cooling followed by no pretreatment (control);
- Group 2 (G2): air polishing with glycine powder (Air Flow Powder Soft 65 μm, pH: 6, EMS; Nyon, Switzerland). Glycine powder was applied with a designated handpiece (Handy2+, EMS) for 10 s on aprismatic enamel with a standardized pressure of ~3.5 bar and a distance of ~10 mm from the surface;
- Group 3 (G3): air polishing with sodium bicarbonate (Air Flow Classic Comfort 40 μm, pH: 8.1, EMS): the powder was applied with a designated handpiece (Handy2+) with the same parameters used in G2 (10 s on aprismatic enamel with a standardized pressure of ~3.5 bar and a distance of ~ 10 mm from the surface);
- Group 4 (G4): airborne-particle abrasion with 50-μm aluminum oxide (aluminum oxide powder (Al_2_O_3_), Kavo; Biberach, Germany). Al_2_O_3_ powder was applied with a designated handpiece (RondoFlex plus 360, Kavo) for 5 s on aprismatic enamel with a standardized pressure of ~2.8 bar and a distance of ~10 mm from the surface;
- Group 5 (G5): extra-fine diamond bur (cod. 862EF.314.010, Komet, Gebr. Brasseler; Lemgo, Germany), mounted on a 1:5 red contra-angle handpiece (RPM: 200,000 working with firm pressure) applied to the superficial surface for 5 s under water irrigation.

After pretreatment, the enamel surfaces were cleaned with air-water spray for 20 s to remove residues. Three subgroups were formed, according to the adhesive procedure (N = 8):

- Subgroup A (SGA): 3-step E&R adhesive (Optibond FL, Kerr; Orange, CA, USA). Application of 37% orthophosphoric acid (Kerr) for 30 s on the enamel surface, followed by adhesive application;
- Subgroup B (SGB): Universal adhesive (Scotchbond Universal, 3M Oral Care; St Paul, MN, USA) in the ER mode. Application of 37% orthophosphoric acid (Kerr) for 15 s on the enamel surface, followed by adhesive application;
- Subgroup C (SGC): Universal adhesive (Scotchbond Universal, 3M Oral Care) in the SE mode. Adhesive application on enamel.

All materials were handled strictly following manufacturers’ instructions.

**Fig 1:**
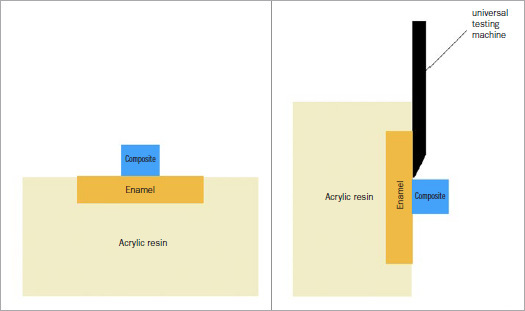
Schematic presentation of specimen preparation for the shear bond strength test. a) Specimens were embedded in acrylic resin and, after surface pretreatments and adhesive applications, resin composite cylinders were built up. b) After 24 h, the specimens were submitted to shear forces with a universal testing machine.

After adhesive polymerization for 20 s with an LED lamp (Valo, Ultradent; South Jordan, UT, USA), a Teflon ring mold (Ø 2.34 mm, 4 mm height, Tesafilm, Tesa SE; Hamburg, Germany) was used to produce resin composite cylinders (Filtek Z 250 XT, 3M Oral Care) over the bonded enamel surface. The mold was filled with 2-mm-thick increments of the nanohybrid composite, and each layer was light cured for 20 s with the same lamp. Excess composite was carefully removed from the matrix with an explorer before final curing. The specimens were stored in distilled water at 37°C before testing (Fig 1).

After 24 h, shear bond strength was measured in a universal testing machine (Instron I model 10/D, Sintech, MTS; Eden Prairie, MN, USA) at a crosshead speed of 0.5 mm/min. In order to measure bond strength, the machine’s chisel-shaped blade was placed at the enamel-composite interface. Bond strengths were recorded in Newtons (N) and converted into MPa. Failure modes were evaluated under an optical microscope at 50X magnification (Discovery V12, Zeiss; Oberkochen, Germany) and classified as adhesive (A), cohesive (C), or mixed (M).

After testing the normality (Shapiro-Wilk test) and homoscedastic (modified Levene’s test) assumptions of the data sets, the bond strength data were analyzed using two-way ANOVA followed by Tukey’s post-hoc test. Fracture modes were statistically evaluated using a chi-squared test. For all statistical tests, the significance level was set at p≤0.05.

### Scanning Electron Microscopy (SEM)

Fifteen sound human molars, extracted for periodontal reasons and stored in saline solution, were selected, cleaned, and inspected for exclusion criteria (cracks, hypoplasia, or white spot lesions).

The teeth were cut into halves. Each half was covered with nail varnish, leaving a 3 x 3-mm area of exposed central enamel. Surface pretreatments were performed on the aprismatic enamel (N of teeth = 3, N of halves = 6 per group), as described for the SBS test.

After pretreatment, half of the specimens were rinsed with water for 20 s and prepared for SEM observation. The second half of the specimens was etched with 37% H_3_PO_4_ for 30 s, rinsed with water for 20 s, and prepared for SEM analysis.

All specimens were ultrasonically cleaned for 2 min. Then, each half was mounted on a metal stub, sputter-coated with gold, and observed under a scanning electron microscope (NovaNanoSEM 450, FEI; Hillsboro, OR, USA) at 5–10 kV and 5000X magnification to qualitatively evaluate the effects of surface treatments on the enamel morphology.

## RESULTS

### Shear Bond Strength Test (SBS)

Means and standard deviations (MPa) of the SBS test for the different groups and subgroups are reported in Table 1.

**Table 1:** SBS means ± standard deviations (expressed in MPa) obtained in the different groups

Pretreatments	Adhesives		
	3-steps E&R	Universal adhesive (E&R)	Universal adhesive (SE)
G1	32.9 ± 7.8^A,b^	38.3 ± 5.5^A,b^	25.2 ± 5.0^B,b^
G2	34.3 ± 5.0^B,b^	41.4 ± 5.9^A,b^	20.7 ± 2.2^C,c^
G3	29.3 ± 2.4^B,b^	43.1 ± 4.0^A,b^	28.1 ± 4.8^B,ab^
G4	42.0 ± 9.1^B,a^	54.2 ± 8.2^A,a^	34.0 ± 3.3^C, a^
G5	30.4 ± 4.9^B,b^	39.3 ± 8.2^A,b^	26.1 ± 6.9^C,b^

Groups with the same superscript letters are not statistically significantly different (p > 0.05). Superscript capital letters indicate differences within the rows; lowercase letters indicate differences within the columns. G1: no pretreatment (control); G2: glycine air polishing; G3: sodium bicarbonate air polishing; G4: sandblasting with Al_2_O_3_; G5: extra-fine bur. SGA: 3-step E&R adhesive (Optibond FL, Kerr); SGB: universal adhesive used in the E&R mode (Scotchbond Universal, 3M Oral Care); SGC: the same universal adhesive used in the SE mode.

The statistical analysis showed a significant difference in SBS for both the variables “pretreatment” (p = 0.01) and “adhesive” (p ≥ 0.003) and for their interaction (p = 0.027).

In terms of pretreatment, Tukey’s post-hoc analysis showed that, irrespective of the adhesive and bonding strategy used, G4 obtained significantly higher SBS compared to the other experimental groups. No differences in bond strength were found between G1, G2, G3, and G5 which attained comparable results (p > 0.05).

In the control group, no differences were found between the 3-step E&R adhesive and the universal adhesive used in E&R mode. However, after enamel surface conditioning, the universal adhesive in E&R mode performed significantly better than did the 3-step E&R and the universal adhesive in SE mode, regardless of the pretreatment method. Additionally, the multistep adhesive achieved higher SBS than did the SE universal adhesive, except in G3, where the two adhesives exhibited comparable results.

Fracture patterns collected for each group and subgroup are reported in Table 2. The chi-squared test showed a significant predominance of adhesive fractures, followed by mixed and cohesive fractures, for all tested groups (p < 0.05).

**Table 2:** Percentages of failure mode among the different groups

Pretreatment	Adhesives								
	3-step E&R			Universal adhesive (E&R)			Universal adhesive (SE)		
	C	A	M	C	A	M	C	A	M
G1	4%	86%	10%	9%	79%	12%	1%	97%	2%
G2	2%	91%	7%	5%	92%	3%	5%	71%	24%
G3	12%	85%	3%	10%	77%	13%	7%	93%	0%
G4	6%	88%	6%	0%	82%	18%	11%	81%	8%
G5	6%	80%	8%	7%	82%	11%	9%	91%	0%

Percentages of failure mode among the different groups (G1: no pre-treatment; G2: glycine air polishing; G3: sodium-bicarbonate air polishing; G4: sandblasting with Al_2_O_3_; G5: extra-fine bur) and subgroups (3-step E&R adhesive [Optibond FL, Kerr]; universal adhesive used in the E&R mode [Scotchbond Universal, 3M Oral Care] and universal adhesive used in the SE mode [Scotchbond Universal, 3M Oral Care]) tested. C: cohesive failure; A: adhesive failure; M: mixed failure. Adhesive failures were predominant in all groups, irrespective of the surface pre-treatments and adhesives.

### SEM Observations

**Fig 2:**
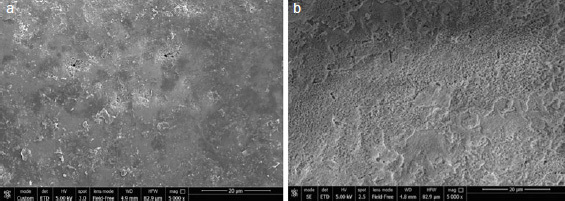
SEM images (5000X) of aprismatic enamel (G1, control) (a) and G1 after conditioning with 37% H_3_PO_4_ for 30 s (b). Wavelike formations typical of aprismatic enamel were observed (a) and they were less consistent after H_3_PO_4_ etching (b).

**Fig 3:**
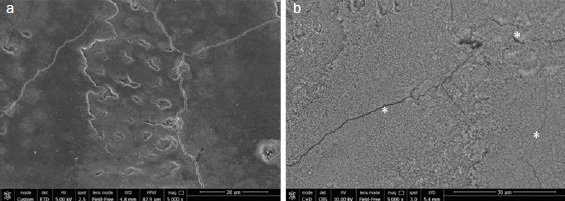
SEM images (5000X) of aprismatic enamel pretreated with glycine air polishing (G2) (a) and G2 after conditioning with 37% H_3_PO_4_ for 30 s (b). Scratches and cracks (white asterisks) were observed after etching (b).

**Fig 4:**
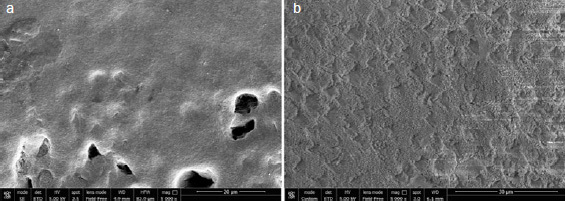
SEM images (5000X) of aprismatic enamel air polished with sodium bicarbonate (G3) (a) and G3 after conditioning with 37% H_3_PO_4_ for 30 s (b).

**Fig 5:**
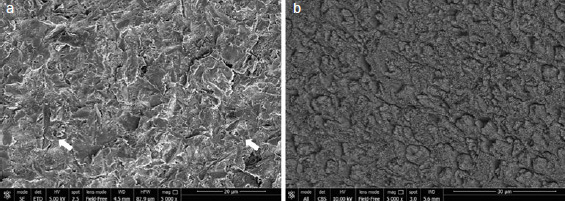
SEM images (5000X) of aprismatic enamel sandblasted with Al_2_O_3_ (G4) (a) and G4 after conditioning with 37% H_3_PO_4_ for 30 s (b). Surfaces appeared rough with protruded edges, interspersed with voids created by the grains of powder (white arrows).

**Fig 6:**
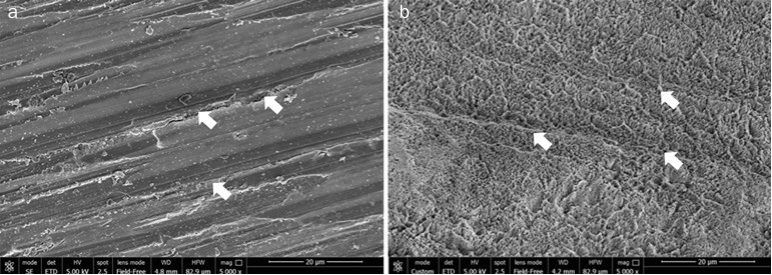
SEM images (5000X) of aprismatic enamel pretreated with an extra-fine bur (G5) (a) and G5 after conditioning with 37% H_3_PO_4_ for 30 s (b). Parallel grooves in the direction of the bur’s movements were observed in both groups (white arrows).

Representative SEM images for each group with/out enamel conditioning with H_3_PO_4_ are presented in Figs 2–6.

Regarding the non-etched sample, the SEM images showed notable differences in enamel surface morphology between the groups. Characteristic depressions with a wavelike appearance in addition to pits and scratches sparsely distributed over the entire surface were present on untreated aprismatic enamel (Fig 2a). The enamel surfaces air polished with glycine and sodium bicarbonate were smoother than the untreated specimens (Figs 3a and 4a, respectively), although the 40-μm sodium bicarbonate grains created more depressions spread along the enamel surface. The analysis of sandblasted specimens revealed the presence of a rough and irregular surface with several elevations of varying heights and rounded edges (Fig 5a). Specimens treated with the extra-fine bur showed several consistent scratches with deep grooves in the direction of the bur’s movement (Fig 6a).

SEM images of the etched control group specimens showed a foamy and inhomogeneous enamel surface. Characteristics of intact enamel, such as pits and scratches, were still detectable on the enamel surfaces (Fig 2b). Pits with slightly rough scratches and several crack lines characterized the glycine air-polished enamel (Fig 3b), while enamel prism boundaries and dissolved core structures were observed in samples treated with sodium bicarbonate (Fig 4b). Airborne-particle abrasion and acid etching produced deep or sharp enamel prism boundaries and dissolved core formations, typical of the type-I etching pattern (Fig 5b). Similar to the sandblasted group, drilling and etching the enamel surface promoted irregularities with a rougher appearance (Fig 6b).

## DISCUSSION

The superficial layer of enamel, best defined as prismless or aprismatic enamel, is a substrate that has the primary characteristic of preventing acid attacks and consequent formation of carious lesions on the tooth. However, resistance to acids also occurs when the aprismatic enamel surface is etched with H_3_PO_4_ or conditioned with an adhesive resin containing acidic monomers, needed for the success of adhesive restorations.^20^

The present in-vitro study aimed at evaluating the effects of different enamel surface pretreatments and bonding strategies on the shear bond strength (SBS) and morphological characteristics of aprismatic enamel. The present results showed a variation in SBS data and enamel texture morphology between the different pretreatments, thus leading to the rejection of the first null hypothesis.

In contrast to deeply and/or diamond-bur prepared enamel substrates, adhesion to superficial enamel has always been more problematic.^1,15^ Indeed, orthodontists are well aware of the problem, as they often use this substrate for cementation of brackets. Furthermore, the high esthetic demands in conservative dentistry today, as well as the minimum-intervention dentistry concept,^11,22^ have emphasized the necessity to find the most efficient protocol for adhesion to aprismatic enamel. At present, only a few studies have been performed on the outcome of bonding to aprismatic enamel pretreated with powders of different compositions and grain sizes, and most of them investigated the clinical outcome of “orthodontic bonding”.^5,17,29^

Several authors have suggested that airborne-particle abrasion produces a homogeneously rough substrate that increases the surface area available for adhesion and the wettability of the enamel.^29,32^ Roughness, resulting from kinetic abrasion, is influenced by the characteristics of the powder: the irregularities are more evident with increasing powder-grain size, as well greater sharpness and hardness of the particles.^26^ This is in accordance with the results obtained in the present study, where the use of 50-µm Al_2_O_3_ before the adhesive procedures resulted in significantly higher bond strength to the aprismatic surface compared to less abrasive powders (such as glycine, sodium bicarbonate, or 25-µm extra-fine bur) or no treatment.

A potential explanation for the lower effectiveness of the softer powders employed in this laboratory study could be their intrinsic composition, which prevents them from modifying and eliminating the non-homogeneous superficial hard and resistant layer of protective enamel from the surface, thus affecting the subsequent application of the adhesive.^23^ Actually, the main indications for use of these prophylaxis powders are the removal of superficial soft biofilm, plaque, and stains, without damaging the enamel surface.^30^ As a matter of fact, different in-vitro studies have evaluated the micromorphological surface characteristics of human dentin and enamel after the application of glycine powder, and they emphasized that it did not produce substantial alteration of human dentin.^13,15^ Also, sodium bicarbonate powders have been proven safe and effective for removing supragingival plaque and stains from intact enamel surfaces without clinically significant surface alterations or substance loss.^23^

Concerning extra-fine bur pretreatment, the data obtained in the present study are lower than those mentioned in the literature for enamel.^7^ However, unlike in the majority of studies testing adhesion to enamel, the diamond bur employed to conduct the present research was an extra-fine bur, which is usually utilized for finishing and polishing of a final restoration, not for the removal of the dental substrate, thus explaining the lack of effectiveness in the modification of aprismatic enamel.

SEM observations confirmed the present findings. Indeed, aluminum oxide seemed to modify the aprismatic surface in a more aggressive and homogeneous way than glycine, bicarbonate, or extra-fine burs, rendering it more similar to the underlying prismatic substrate, thus corroborating the results of the SBS data.

In addition, as opposed to no treatment, airborne-particle abrasion, and the use of an extra-fine bur, the pretreatment with bicarbonate and glycine seemed to have a somewhat deleterious effect on H_3_PO_4_ etching effectiveness, as only small portions of prism perimeters were exposed. Alternatively, when alumina powder and an extra-fine bur were used, more aprismatic enamel was removed, and the etching gel was able to expose prisms homogeneously and predictably.

However, the roughness and homogeneity of the substrate are not the only factors influencing bond strength. The mechanical and physical properties of the adhesive resin (eg, wettability and the molecules included in the formulation) and the bonding protocol used are of high importance.^16,41^

To investigate the potential effects of various adhesives on aprismatic enamel, the present study evaluated bond strengths of samples treated with different adhesive systems and protocols. The universal adhesive in ER mode performed better than the same adhesive applied in SE mode, independent of the pretreatment performed. In addition, the 3-step ER adhesive also yielded higher SBS than the universal adhesive in SE mode, leading to the rejection of the second null hypothesis.

These findings are in accordance with numerous reports in the literature that claim the superiority of phosphoric acid application before adhesion on enamel, independently of the characteristics of the substrate.^7,10^ Indeed, the demineralization of the highly mineralized enamel with a strong acid is considered the “gold standard” for the achievement of an ideal substrate for adhesion.^10^

Acid etching promotes demineralization, selectively dissolves the enamel rods, and creates microporosities, which are penetrated by bonding agents via capillary attraction.^16^ Micromechanical interlocking of tiny resin tags within the acid-etched enamel surface provides the best achievable bond to the dental substrate.^41^

In the present study, the 3-step ER adhesive was shown to be less effective than the universal adhesive employed in ER mode, especially after air polishing with sodium bicarbonate, even though this category of adhesives is considered the “gold standard”.^4^ The present findings may be due to the chemical and mechanical changes to the dental substrate caused by air abrasion with polishing powders (glycine or sodium bicarbonate), which could prevent the penetration of the adhesive resin.^19,30,40^ Sodium bicarbonate (pH 8.3) and glycine (pH 6.0) may have buffered the conditioning gel reducing its demineralizing potential, consequently reducing the bonding performance.^27^ The buffering effect of the polishing powder could be limited when adhesion is performed with a universal adhesive in ER mode, in which a double application of acidic molecules on the enamel surface is performed. Firstly, phosphoric acid is applied and rinsed away with water after 15 s; secondly, adhesive resin containing acidic monomers is applied to the surface, counteracting the buffering potential of the polishing powders. Furthermore, the universal adhesive employed in this study is less viscous than the 3-step ER adhesive, and this characteristic increases the surface wettability and improves the ability of the adhesive resin to infiltrate enamel.^3^ It should also be emphasized that several clinical approaches have been proposed to further enhance the enamel bond strength, independent of the viscosity of the material, such as increasing the adhesive application time and actively scrubbing the adhesive on the dental surface.^8,9^ These methods have been previously reported to facilitate the diffusion of the acidic monomers of bonding systems, thus producing a more retentive pattern on enamel.

Regarding SE mode, the results of the present study confirm previous findings.^6,18,31^ The adhesion to enamel without the phosphoric acid etching step is considered less effective and unable to fully demineralize and impregnate the enamel surface.^6,18,31^ The universal adhesive employed in SE mode proved to be less effective than the same adhesive in ER mode or the classic 3-step ER adhesive. Indeed, it has been reported that enamel etching with phosphoric acid yields approximately 33% more surface free energy than without pre-etching, thus providing a possible explanation for the data reported here.^25^

Further studies are necessary to evaluate the effects of the different enamel surface treatments on the longevity of the bond.

## CONCLUSIONS

Shear bond strength to aprismatic enamel is increased by surface airborne-particle abrasion with aluminum oxide followed by phosphoric acid application, particularly in combination with a universal adhesive in the etch-and-rinse mode.

Therefore, a more effective surface pretreatment and etching should be performed when preparing the tooth surface for the non-prep esthetic restorations. Furthermore, glycine and sodium bicarbonate air-polishing powders were unable to modify the aprismatic enamel surface.

## Clinical relevance:

Aprismatic enamel is a substrate that prevents acid attacks. However, resistance to acids also occurs when the surface is etched with H_3_PO_4_ or conditioned with the acid present in the adhesive. Thus, an effective surface pretreatment should be performed when preparing the tooth surface for restorations.

## References

[ref1] Arakawa Y, Takahashi Y, Sebata M (1979). The effect of acid etching on the cervical region of the buccal surface of the human premolar, with special reference to direct bonding techniques. Am J Orthod.

[ref2] Asmussen E, Peutzfeldt A (2005). Resin composites: Strength of the bond to dentin versus surface energy parameters. Dent Mater J.

[ref3] Asmussen E, Peutzfeldt A (1998). Surface energy characteristics of adhesive monomers. Dent Mater J.

[ref4] Bedran-Russo A, Leme-Kraus AA, Vidal CMP, Teixeira EC (2017). An overview of dental adhesive systems and the dynamic tooth-adhesive interface. Dent Clin North Am.

[ref5] Borsatto MC, Catirse AB, Palma Dibb RG, Nascimento TN, Rocha RA, Corona SA (2002). Shear bond strength of enamel surface treated with air-abrasive system. Braz Dent J.

[ref6] Bracket WW, Ito S, Nishitani Y, Hais- ch LD, Pashley DH (2006). The microtensile bond strength of self-etch adhesives to ground enamel. Oper Dent.

[ref7] Breschi L, Mazzoni A, Ruggeri A, Cadenaro M, Di Lenarda R, De Stefano Dorigo E (2008). Dental adhesion review: Aging and stability of the bonded interface. Dent Mater.

[ref8] Cardenas AM, Siqueira F, Rocha J, Szesz AL, Anwar M, El-Askary F, Reis A, Loguercio A (2016). Influence of conditioning time of universal adhesives on adhesive properties and enamel-etching pattern. Oper Dent.

[ref9] Cardenas AFM, Armas-Veja A, Rodriguez Villarreal JP, Siqueira FSF, Muniz LP, Campos VS, Reis A, Loguercio AD (2019). Influence of the mode of application of universal adhesive systems on adhesive properties to fluorotic enamel. Braz Oral Res.

[ref10] Cardoso MV, de Almeida Neves A, Mine A, Coutinho E, Van Landuyt K, De Munck J, Van Meerbeek B (2011). Current aspects on bonding effectiveness and stability in adhesive dentistry. Aust Dent J.

[ref11] Comba A, Vergano EA, Baldi A, Alovisi M, Pasqualini D, Castroflorio T, Stura I, Migliaretti G, Berutti E, Scotti N (2021). 5-year retrospective evaluation of direct composite restorations in orthodontically treated patients. J Dent.

[ref12] Faul F, Erdfelder E, Lang AG, Buchner A (2007). G*Power 3: a flexible statistical power analysis program for the social, behavioral, and biomedical sciences. Behav Res Methods.

[ref13] Frankenberger R, Lohbauer U, Tay FR, Taschner M, Nikolaenko SA (2007). The effect of different air-polishing powders on dentin bonding. J Adhes Dent.

[ref14] Galloway SE, Pashley DH (1987). Rate of removal of root structure by the use of the Prophy-Jet device. J Periodontol.

[ref15] Gwinnett AJ, Matsui A (1967). A study of enamel adhesives. The physical relationship between enamel and adhesive. Arch Oral Biol.

[ref16] Gwinnett AJ (1981). Acid etching for composite resins. Dent Clin North Am.

[ref17] Halpern RM, Rouleau T (2010). The effect of air abrasion preparation on the shear bond strength of an orthodontic bracket bonded to enamel. Eur J Orthod.

[ref18] Hannig M, Bock H, Bott B, Hoth-Hannig W (2002). Inter-crystallite nanoretention of self-etching adhesives at enamel imaged by transmission electron microscopy. Eur J Oral Sci.

[ref19] Janiszewska-Olszowska J, Drozdzik A, Tandecka K, Grocholewicz K (2020). Effect of air-polishing on surface roughness of composite dental restorative material –comparison of three different air-polishing powders. BMC Oral Health.

[ref20] Kodaka T, Higashi S (1995). Fine incremental lines observed on the enamel surfaces with a aprismatic structure in human teeth after phosphoric acid etching. Kaibogaku Zasshi.

[ref21] Kodaka T, Kuroiwa M, Higashi S (1991). Structural and distribution patterns of surface ‘aprismatic’ enamel in human permanent teeth. Caries Res.

[ref22] Lee JY, Watt RG, Williams DM, Giannobile WV (2017). A new definition for oral health: Implications for clinical practice, policy, and research. J Dent Res.

[ref23] Mahlendorff M (1989). Evaluation of the relationships between abrasion and surface alterations after professional tooth cleaning. Deutsche Zahnärztliche Zeitschrift.

[ref24] Maravic T, Mazzoni A, Comba A, Scotti N, Checchi V, Breschi L (2017). How stable is dentin as a substrate for bonding?. Curr Oral Health Rep.

[ref25] Nagura Y, Tsujimoto A, Barkmeier WW, Watanabe H, Johnson WW, Takamizawa T, Latta MA, Miyazaki M (2018). Relationship between enamel bond fatigue durability and surface free-energy characteristics with universal adhesives. Eur J Oral Sci.

[ref26] Neme AL, Frazier KB, Roeder LB, Debner TL (2002). Effect of prophylactic polishing protocols on the surface roughness of esthetic restorative materials. Oper Dent.

[ref27] Nikaido T, Kataumi M, Burrow MF, Inokoshi S, Yamada T, Takatsu T (1996). Bond strengths of resin to enamel and dentin treated with low-pressure air abrasion. Oper Dent.

[ref28] Pashley DH (1996). Dynamics of the pulpo-dentin complex. Crit Rev Oral Biol Med.

[ref29] Patcas R, Zinelis S, Eliades G, Eliades T (2015). Surface and interfacial analysis of sandblasted and acid-etched enamel for bonding orthodontic adhesives. Am J Orthod Dentofacial Orthop.

[ref30] Pelka MA, Altmaier K, Petschelt A, Lohbauer U (2010). The effect of air-polishing abrasives on wear of direct restoration materials and sealant. J Am Dent Assoc.

[ref31] Perdigão J, Geraldeli S (2003). Bonding characteristics of self-etching adhesives to intact versus prepared enamel. J Esthet Restor Dent.

[ref32] Roeder LB, Berry EA 3rd, You C, Powers JM (1995). Bond strength of composite to air-abraded enamel and dentin. Oper Dent.

[ref33] Schneider PM, Messer LB, Douglas WH (1981). The effect of enamel surface reduction in vitro on the bonding of composite resin to permanent human enamel. J Dent Res.

[ref34] Scotti N, Cavalli G, Gagliani M, Breschi L (2017). New adhesives and bonding techniques Why and when?. Int J Esthet Dent.

[ref35] Scotti N, Comba A, Gambino A, Manzon E, Breschi L, Paolino D, Pasqualini D, Berutti E (2016). Influence of operator experience on non-carious cervical lesion restorations: Clinical evaluation with different adhesive systems. Am J Dent.

[ref36] Scotti N, Comba A, Gambino A, Paolino DS, Alovisi M, Pasqualini D, Berutti E (2014). Microleakage at enamel and dentin margins with a bulk fills flowable resin. Eur J Dent.

[ref37] Sofan E, Sofan A, Palaia G, Tenore G, Romeo U, Migliau G (2017). Classification review of dental adhesive systems: from the IV generation to the universal type. Ann Stomatol (Roma).

[ref38] Stacey GD (1993). A shear stress analysis of the bonding of porcelain veneers to enamel. J Prosthet Dent.

[ref39] Strassler HE (2007). Minimally invasive porcelain veneers: indications for a conservative esthetic dentistry treatment modality. Gen Dent.

[ref40] Tamura Y, Takamizawa T, Shimamura Y, Akiba S, Yabuki C, Imai A, Tsujimoto A, Kurokawa H, Miyazaki M (2017). Influence of air-powder polishing on bond strength and surface-free energy of universal adhesive systems. Dent Mater J.

[ref41] Van Meerbeek B, De Munck J, Mattar D, Van Landuyt K, Lambrechts P (2003). Microtensile bond strengths of an etch&rinse and self-etch adhesive to enamel and dentin as a function of surface treatment. Oper Dent.

[ref42] Whittaker DK (1982). Structural variations in the surface zone of human tooth enamel observed by scanning electron microscopy. Arch Oral Biol.

